# Effects of specialized proresolving mediators on gut epithelial barrier in early life

**DOI:** 10.1016/j.jpet.2026.103815

**Published:** 2026-01-21

**Authors:** Jing Chen, Sarah Ouahoud, Renee R.C.E. Schreurs, Sander Meisner, Jacqueline L.M. Vermeulen, Manon E. Wildenberg, Wouter J. de Jonge, Johannes B. van Goudoever, Tim G.J. de Meij, Vanesa Muncan, Chris H.P. van den Akker

**Affiliations:** 1 Tytgat Institute for Liver and Intestine Research and Department of Pediatrics, Amsterdam UMC, Emma Children’s Hospital, Amsterdam, The Netherlands; 2 Amsterdam Gastroenterology Endocrinology and Metabolism (AGEM) Research Institute, Amsterdam, The Netherlands; 3 Amsterdam Reproduction & Development (AR&D) Research Institute, Amsterdam, The Netherlands; 4 Department of Pediatrics – Neonatology, Amsterdam UMC, Emma Children’s Hospital, University of Amsterdam, Amsterdam, The Netherlands; 5 Department of Experimental immunology, Amsterdam UMC, Emma Children’s Hospital, University of Amsterdam, Amsterdam, The Netherlands; 6 Department of Pediatric Gastroenterology, Amsterdam UMC, Emma Children’s Hospital, University of Amsterdam, Amsterdam, The Netherlands

**Keywords:** Necrotizing enterocolitis, Preterm infants, Organoid, Specialized proresolving mediator

## Abstract

Damage to the intestinal epithelial barrier is a hallmark of inflammatory diseases such as necrotizing enterocolitis. Specialized proresolving mediators (SPMs), such as lipoxin A4, resolvin D1, and resolvin E1, which are derived from essential fatty acids, have been shown to aid in resolving inflammation and promote mucosal healing. This study aimed to explore the effects of specific SPMs on intestinal inflammatory response in an early life in vitro model. We established 3-dimensional and 3-dimensional organoid cultures from fetal and pediatric intestines and investigated the effect of an SPM cocktail (lipoxin A4, resolvin D1, and resolvin E1) on gut epithelial maturation and barrier function. An inflammatory response of the gut barrier was provoked by lipopolysaccharide and flagellin stimulations combined with proinflammatory cytokines, tumor necrosis factor-*α*, and interferon gamma. Additionally, repetitive mechanical wounding was developed to test the effects of the SPM cocktail on 2-dimensional organoid monolayers. Under physiological conditions, we observed no effect of SPM cocktail treatment on gut epithelial maturation. Upon cytokine challenge, there was no modulation of the inflammatory tone of the gut barrier by the SPM cocktail. However, during the repetitive wounding and recovery assay, SPM cocktail treatment accelerated barrier recovery and maintained barrier integrity for 24 hours after repeated injuries. Our findings suggest that the SPM cocktail does not affect bacterial product- or cytokine-induced epithelial inflammation, although it may accelerate epithelial barrier recovery in mechanically wounded monolayers. These results provide valuable insights into the therapeutic potential of SPMs in neonatal intestinal inflammation.

**Significance Statement:**

Using early life intestinal organoid models, we found that although specialized proresolving mediators did not alter cytokine- or bacterial product-induced inflammation, they significantly enhanced epithelial barrier recovery following repeated mechanical injury.

## Graphical abstract

**Figure:**
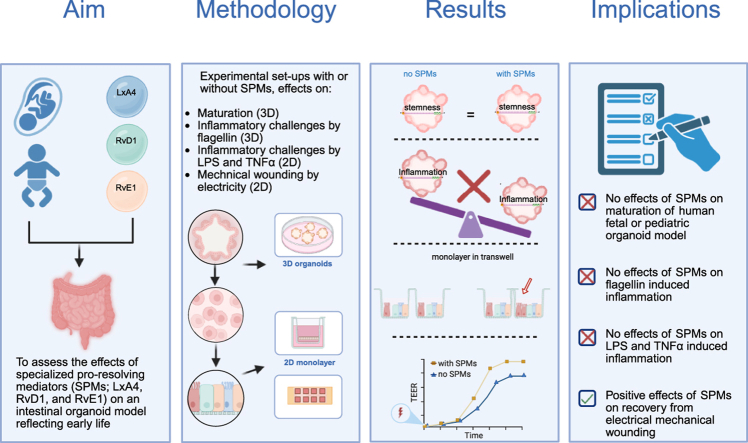

## 1 Introduction

Damage to epithelial cells can result from acute and chronic inflammatory conditions.^1^ In preterm infants, the most common acute gastrointestinal inflammatory disease is necrotizing enterocolitis (NEC).^2^ The pathogenesis of NEC is not completely understood, but is considered multifactorial, and the onset of NEC is characterized by impaired epithelial barrier integrity, which facilitates the invasion of luminal antigens and pathogens, thereby triggering an inflammatory response.^3^ Mechanistically, toll-like receptor 4 is involved in NEC pathogenesis and is activated by microbes such as *Escherichia coli*.^4^ This toll-like receptor 4 activation triggers inflammatory responses and contributes to the upregulation of key inflammatory mediators, including tumor necrosis factor-*α* (TNF*α*), interleukin (IL)-6, IL-8, and interferon gamma (IFNγ).^4^^,^^5^ Infants who are fed with human milk have a lower chance of developing NEC compared to formula-fed infants.^6^ This protective role is not fully elucidated; however, several factors from human milk have been studied for anti-inflammatory properties.^6^^,^^7^ Human milk is most known for its bioactive proteins such as lactoferrin and immunoglobulins, but milk lipids are also getting more attention because of the direct antimicrobial and overall immunomodulatory properties of specific fatty acid derivatives, so-called oxylipins.^8^^,^^9^ These bioactive oxylipins, derived from *ω*-6 and *ω*-3 fatty acids, include families such as lipoxins, resolvins, protectins, and maresins.^10^^,^^11^ Collectively, these are called specialized proresolving mediators (SPMs). Human milk contains SPMs in high concentrations, albeit they are also synthesized locally in the gut by immune and epithelial cells.^12^^,^^13^ SPMs are known to help maintain intestinal mucosal homeostasis by playing a crucial role in resolving intestinal mucosal inflammation.^13^, ^14^, ^15^, ^16^, ^17^ For instance, exposure of colonic tissue to lipoxin A4 (LxA4) attenuates TNF*α*-mediated epithelial cell apoptosis, thus protecting the mucosa against TNF*α*-induced damage.^16^ In addition, it is demonstrated that resolvin D1 (RvD1) significantly attenuates small intestinal damage induced by indomethacin through inhibiting gene expression levels of TNF*α* and IL1*β*.^17^ Similarly, resolvin E1 (RvE1) promotes mucosal healing by enhancing intestinal epithelial cell migration and proliferation.^13^ Therefore, exogenously administered SPMs may have a therapeutic role in resolving inflammation and promoting mucosal healing.

In this study, we investigated whether exogenously administered SPMs could aid the resolution of induced damage to gut epithelial cells during early life. During this period of life, the gut epithelium undergoes significant functional and metabolic changes and gradually matures to adult epithelium.^18^ To recapitulate this early life period, we used fetal and pediatric intestinal tissues to generate organoid cultures. Organoid cultures are initially 3-dimensional (3D) structures resembling the gut by containing the specific cell types of the epithelium with functional characteristics similar to those observed in vivo. In recent years, it has been shown that organoids retain tissue characteristics, including regional identity of the gastrointestinal tract as well as developmental and/or disease stage, and hence are excellent in vitro models to study gut physiology and disease.^19^^,^^20^ Additionally, when cultured as 2-dimensional (2D) epithelial monolayers, gut organoids are suitable for studying the properties and functions of the intestinal epithelial barrier.^19^

We hypothesize that SPMs have the potential to alleviate intestinal inflammation and restore the gut barrier. To explore this, we treated human gut organoids with various stimuli to induce epithelial damage and an SPM cocktail consisting of LxA4, RvD1, and RvE1. The subsequent effects of the SPM cocktail on the intestinal epithelium were measured, focusing on maturation, resolution of inflammation, and repair of barrier function.

## 2 Material and methods

### 2.1 Human donors

Human fetal intestine tissue samples (gestational age 17–20 weeks, both proximal and distal intestine) were obtained by the Human Immune System Mouse Facility of the Amsterdam University Medical Center in The Netherlands. All material was collected from donors from whom written informed consent had been obtained for the use of collected tissue for research purposes. Human pediatric intestinal tissue samples (proximal and/or distal intestine) from infants aged 2 weeks up to 5 months were obtained from pediatric bowel surgeries of stoma reversal at the pediatric surgery department of our hospital. All pediatric patients had received antibiotics (cefazolin and metronidazole) 30 minutes before surgery. Ethical approval was obtained from the ethical committee of the Amsterdam UMC, under approval number: W16_161 #16.188.

All samples were coded to protect patient confidentiality. Written informed consent was obtained from all individual participants or their legal guardians. The collection and use of patient intestinal tissue samples in this study were carried out in compliance with applicable laws and regulations, as outlined in the Amsterdam University Medical Center research code, within a certified laboratory accredited under ISO15189 (M304).

### 2.2 3D human fetal intestinal organoid culture

Human fetal intestinal organoid (HFO) cultures were isolated from fetal intestine as described previously.^21^ In short, after identification of appendix anatomy, human fetal small intestinal tissue was cut open longitudinally, cut into pieces of ∼5 mm, and extensively washed with cold PBS. Next, the tissue was incubated in 5 mM EDTA (Invitrogen, cat#15575-038) and 2 mM DL-dithiothreitol (Sigma, cat#D9779-5G) in PBS for 60 minutes at 4 °C on a shaker (400–600 rpm). After the incubation, crypts were mechanically detached from the tissue, and the supernatant was collected and subsequently passed through a cell strainer (70 *μ*m) and centrifuged at 950 rpm for 10 minutes at 4 °C. Then, pellets were washed in cold advanced Dulbecco’s modified Eagle medium F12 1:1 (Invitrogen, cat#12634028) supplemented with 100 U-mg/mL penicillin/streptomycin (Invitrogen, cat#15140122), 10 mM HEPES (Life Technologies, cat#15630-056), and 1× Glutamax (Invitrogen/Gibco, cat#35050-038). Isolated crypts were resuspended in Matrigel (Corning, cat#356231), dispensed in three 10 *μ*L droplets per well in a 24-well tissue culture plate, covered with 500 *μ*L medium, and incubated at 37 °C for 15 minutes. Organoid cultures were maintained in Human IntestiCult Organoid Growth Medium (HIOGM, Stemcell Technologies, cat#100-0190) supplemented with again 100 U-mg/mL penicillin–streptomycin (Gibco, Thermo Fischer Scientific, cat#15140122) and incubated at 37 °C under 5% CO_2_. Medium was refreshed every 3 days, and organoids were passaged by mechanical disruption every 6–7 days, or by enzymatic dissociation using TrypLE as described elsewhere.^22^ In experiments requiring the cells to reflect the mature intestine, culture medium was switched from HIOGM to Human IntestiCult Organoid Differentiation Medium (HIODM, Stemcell Technologies, cat#100-0212) when treatment started.

### 2.3 3D human pediatric intestinal organoid culture

Pediatric intestinal specimens were sectioned and opened longitudinally, followed by rinsing in ice-cold PBS. The mucosa was carefully separated from the submucosa, and the mucosal tissue was cut into approximately 5-mm fragments. These fragments were incubated for 1 hour at 4 °C in a solution of 0.5 M EDTA with 1 M DTT in Dulbecco’s modified Eagle medium-12. After incubation, the isolation of human pediatric intestinal organoids (HPOs) from the mucosal tissue was performed as described for the human fetal tissue.

### 2.4 SPM preparation and handling

LxA4, RvD1, and RvE1 were obtained from Cayman Chemical (item numbers 90410, 10012554, and 10007848; batch numbers 0614337-35, 0471528-244, and 0659583-2, respectively). Compounds were supplied predissolved in ethanol (25 *μ*g per vial). Upon receipt, each vial was aliquoted into 500 ng portions (5–10 *μ*L per aliquot, depending on the supplied volume) without further dilution, following the manufacturer’s instructions. Aliquots were stored at −80 °C protected from light and thawed on ice immediately before use. Stock solutions were not subjected to more than 2 freeze-thaw cycles. Working solutions were prepared fresh for each experiment by diluting stocks in culture medium to achieve final concentrations of 100, 200, or 500 nM. Vehicle control conditions received equivalent volumes of ethanol (final concentration < 0.3% v/v in all conditions). All SPM-containing media were protected from light during incubation.

### 2.5 3D organoid cultures

#### 2.5.1 SPM treatment without noxious stimulation

Dose selection for the current study was guided by the low-nanomolar affinities of LxA4, RvD1, and RvE1 for their cognate receptors and by prior intestinal epithelial studies describing 10–500 nM SPMs for wound repair exploration in vitro.^12^^,^^13^^,^^23^, ^24^, ^25^

3D HFO and HPO cultures from each independent donor were treated for 72 hours after passaging with either HIODM alone (control group) or together with a cocktail of 3 SPMs, namely LxA4, RvD1, and RvE1, each at concentrations of either 100, 200, or 500 nM to determine optimal effectiveness. Total RNA was collected 72 hours later for further analyses.

#### 2.5.2 Bacterial protein stimulation

To induce inflammation, HFO cultures were stimulated with lipopolysaccharide (LPS) at a concentration of either 100 or 200 *μ*g/mL and/or flagellin (FL) at 100 ng/mL for 24 hours. Following stimulation, both supernatant and total RNA were harvested for further analysis.

To assess the effect of SPMs on inflammation, 3D HFOs were treated with an SPM cocktail (LxA4, RvD1, and RvE1 at a concentration of 200 nM each) 1 hour prior to stimulation with FL at a concentration of 100 ng/mL. Supernatant and total RNA were collected 24 hours after stimulation for analysis.

#### 2.5.3 2D monolayer generation and transepithelial electrical resistance measurements

2D monolayer HFO and HPO cultures were generated according to an established protocol^19^ and seeded on transwell cell culture inserts (0.4-*μ*m pore size, Cell Quart, cat#9320414) that were coated with 100 *μ*L of 20 *μ*g/mL rat tail collagen type I (Ibidi) dissolved in 0.1% (v/v) acetic acid for 1 hour at room temperature and rinsed with PBS twice prior to use. HFO and HPO cultures were expanded through multiple passages and harvested on day 6. Single-cell suspension was achieved by treating the organoids with TrypLE (Invitrogen) for 10 minutes at 37 °C. Cells were then diluted to a concentration of 1 × 10^6^ cells/mL, and 100 *μ*L of cell suspension per insert was seeded (=1.5 × 10^5^ cells per insert), and maintained in HIOGM containing Y-27632 (Sigma, cat#Y0503-5MG), with 100 *μ*L in the apical compartment and 600 *μ*L in the basolateral compartment, for the first 3 days of culture. The culture medium was switched from HIOGM to HIODM on day 7 post-transwell seeding, corresponding to the time point at which a fully established 2D monolayer was observed (Supplemental Fig. 1). HIODM was maintained for the remainder of the experiment. Monolayer formation was confirmed by plateauing transepithelial electrical resistance (TEER) values and microscopic evaluation.

Monolayer confluence and differentiation, as well as the barrier integrity, were assessed by measuring TEER using an *Ω*-meter (EVOM2; World Precision Instruments, serial#195696), following the manufacturer's protocol. Each insert underwent 2 measurements (once in each pore), and the average values were adjusted for background TEER and surface area of the insert to determine the net-area resistance in Ω∗cm^2^.

#### 2.5.4 Bacterial protein and cytokine stimulation of 2D organoid monolayers

Before the administration of noxious stimulators, monolayers were preincubated with an SPM cocktail containing LxA4, RvD1, and RvE1 at a concentration of 200 nM each for 1 hour. To assess the barrier function of 2D intestinal organoid monolayers, recombinant human (rh)TNF*α* (400 ng/mL) alone or combined with bacterial protein LPS (250 *μ*g/mL), or a cytokine mix containing rhIFN*γ* at 50 ng/mL together with rhTNF*α* at 200 ng/mL, was applied to induce inflammation and disrupt intestinal barrier integrity. The monolayers were then incubated for 6 days, with either media alone or media with the SPM cocktail. Both conditions were refreshed on the third day. TEER values were measured every 3 days to evaluate barrier function (Supplemental Fig. 1).

The concentrations of inflammatory stimuli used in this study (LPS 100–250 *μ*g/mL, FL 100 ng/mL, rhTNF*α* 200–400 ng/mL, and rhIFN*γ* 50 ng/mL) were selected on the basis of published protocols for inducing barrier disruption in intestinal epithelial cell cultures and organoid models.^26^, ^27^, ^28^, ^29^, ^30^ These concentrations represent relatively strong inflammatory challenges designed to consistently induce measurable barrier dysfunction across donors.

#### 2.5.5 Repetitive wounding and recovery assay

In order to assess the ability of intestinal organoid cultures to recover from repetitive wounding experiments with or without pretreatment with SPM cocktail, we used an electric cell-substrate impedance sensing (ECIS) Z-*θ* (Applied Biophysics). ECIS is a real time, label-free, impedance-based method used to study various cell behaviors, such as cell migration and barrier function in vitro. ECIS arrays were prepared as previously described.^31^ In brief, 8W10E ECIS arrays were pretreated with 10 mM l-cysteine for 30 minutes prior to application of coating with 100 *μ*L of 20 *μ*g/mL rat tail collagen type I (Ibidi) dissolved in 0.01% (v/v) acetic acid for 1 hour at room temperature.

HFO and HPO cultures were dissociated into single cells and subsequently seeded at a density of 2.0 × 10^5^ cells per well. In this study, ECIS was used to develop a repetitive wounding and recovery model, during which TEER was measured in real time. Confluent epithelial monolayers were pretreated with an SPM cocktail (containing LxA4, RvD1, and RvE1 at a concentration of 200 nM each) or with simple medium refreshment. After 60 minutes, the monolayers were exposed to a current of 4000 *μ*A at a frequency of 50 kHz for 30 seconds, after which the cultures were allowed to recover. Once the TEER value recovered to 70% of its original maximal value, another electrical stimulation was administered. This protocol was repeated to generate the third, fourth, and fifth wounds. These sequential wounding cycles were necessary due to the resilience of our cultures and allowed us to mimic the repeated epithelial insults characteristic of neonatal intestinal injury while assessing the sustained capacity of SPMs to promote recovery. TEER measurements were recorded every 5 minutes at a frequency of 500 Hz over approximately 40 hours in total (Supplemental Fig. 2).

### 2.6 RNA isolation and Reverse Transcription-quantitative PCR

RNA was isolated using the Bioline ISOLATE II RNA Mini kit (BIO-52073, Bioline) according to manufacturers' protocol. cDNA synthesis was performed following a laboratory-specific protocol, diverging from the manufacturer's instructions. Quantitative Reverse Transcription-quantitative PCR (RT-qPCR) analysis was conducted using a Bio-Rad iCycler system and the SensiFast SYBR No-ROX Kit (GC-biotech Bio-98020), following the guidelines provided by the manufacturer with adaptations tailored to our laboratory's specifications. From a panel of 5 genes—glyceraldehyde-3-phosphate dehydrogenase, *36B4*, β-actin, cyclophilin, and hypoxanthine phosphoribosyltransferase—the most stable reference genes were identified using GeNorm. Relative expression levels with N0 values were obtained using LinRegPCR software application and normalized to reference genes. All qPCR primer pairs used in this study were prevalidated by the institutional core facility and confirmed to perform within acceptable efficiency and specificity ranges. Primer sequences are listed in Supplemental Table 1.

### 2.7 Cytometric beads assay

To measure inflammatory responses of the HFO cultures, cytokine levels in the medium collected from the 3D assay of FL-induced inflammation were quantified using the BD Cytometric Bead Array (BD Biosciences) for human inflammatory cytokines, according to the manufacturer's instructions. Subsequently, cytokines are acquired by flow cytometry (FACSDiva, BD Bioscience). Data were analyzed using FlowJo software.

### 2.8 Statistical analysis

**Fig. 1:**
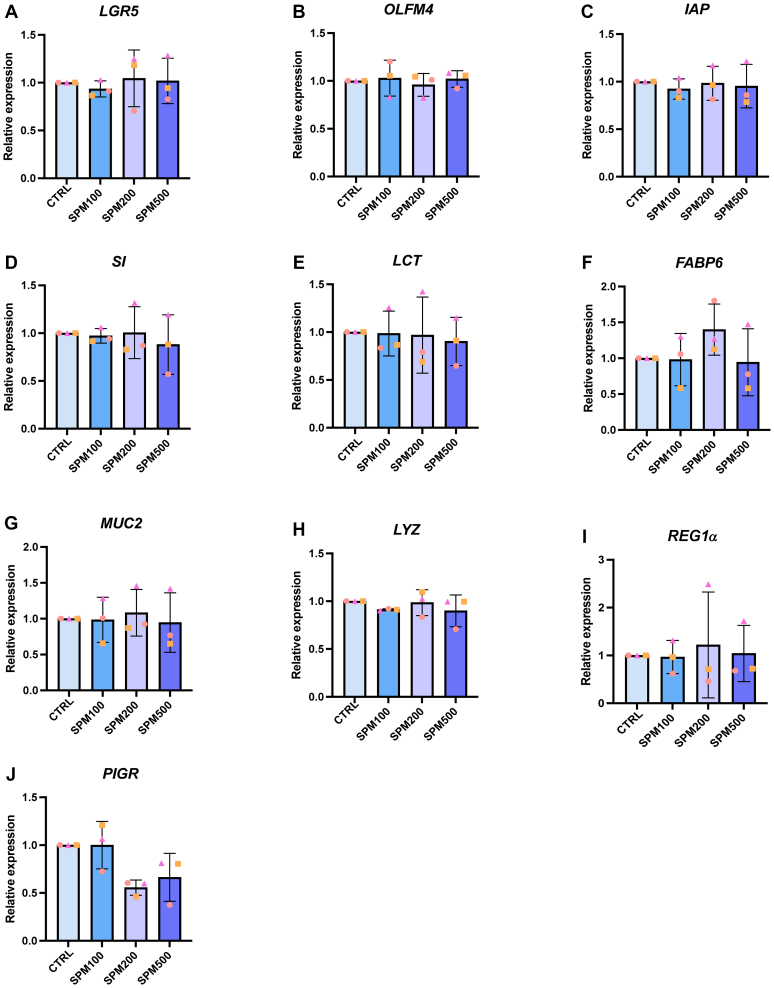
Bar graphs of relative expression for various differentiation and maturation markers (RT-qPCR) in HFO cultures (proximal gut) when treated with an SPM cocktail in various concentrations (100, 200, or 500 nM) for 72 hours in comparison to the control group (CTRL). Stemness was assessed by (A) leucine-rich repeat-containing G-protein coupled receptor 5 (*LGR5*) and (B) olfactomedin 4 (*OLFM4*); brush-border enzyme and absorption markers by (C) intestinal alkaline phosphatase (*IAP*), (D) sucrase–isomaltase (*SI*), (E) lactase (*LCT*), and (F) fatty acid-binding protein 6 (*FABP6*); goblet cells by (G) mucin 2 (*MUC2)*; Paneth cells by (H) iysozyme (*LYZ*) and (I) retinoic acid–inducible gene 1 *α* (*REG1α*); and defense markers by (J) polymeric immunoglobulin receptor (). Three technical replicates per donor were averaged prior to analysis. Values are mean ± SD of *n* = 3 independent donors per condition. Similar symbols represent the same donor across conditions. No significant differences were observed between treatment groups as determined by the Friedman test, followed by Dunn post hoc test with Bonferroni correction for comparisons to the control.

**Fig. 2:**
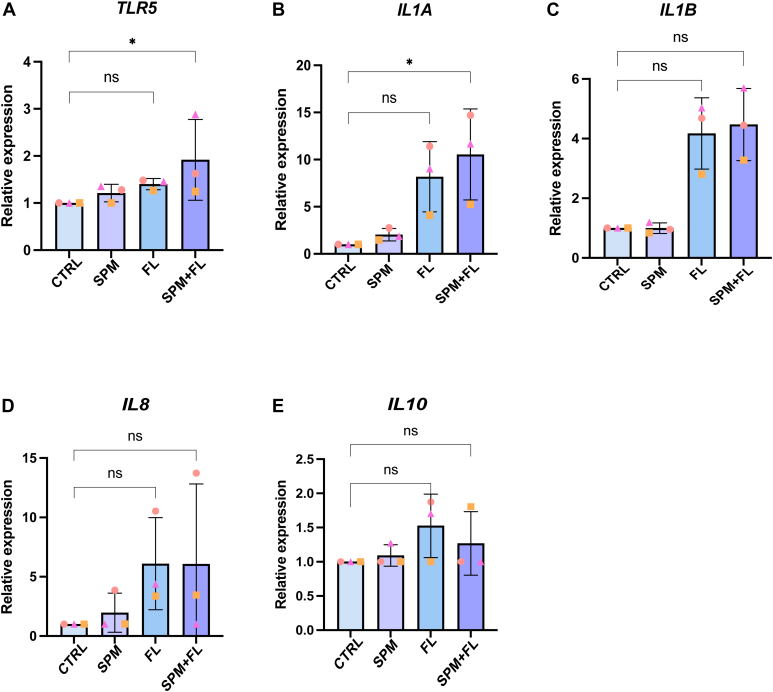
Bar graphs of relative gene expression levels (RT-qPCR) of (A) *TLR5*, (B) *IL1α*, (C) *IL1β*, (D) *IL8*, and (E) *IL10* in 3D HFO cultures (distal intestine) without stimulation (control group [CTRL]), treated with the SPM cocktail at 200 nM, treated with FL at 100 ng/mL, and treated with both SPM cocktail and FL. Three technical replicates per donor were averaged prior to analysis. Values are mean ± SD of *n* = 3 independent donors per condition. Similar symbols represent the same donor across conditions. ∗*P* < .05; ns denotes no significant differences were observed between treatment groups as determined by the Friedman test, followed by Dunn post hoc test with Bonferroni correction for comparisons to control.

**Fig. 3:**
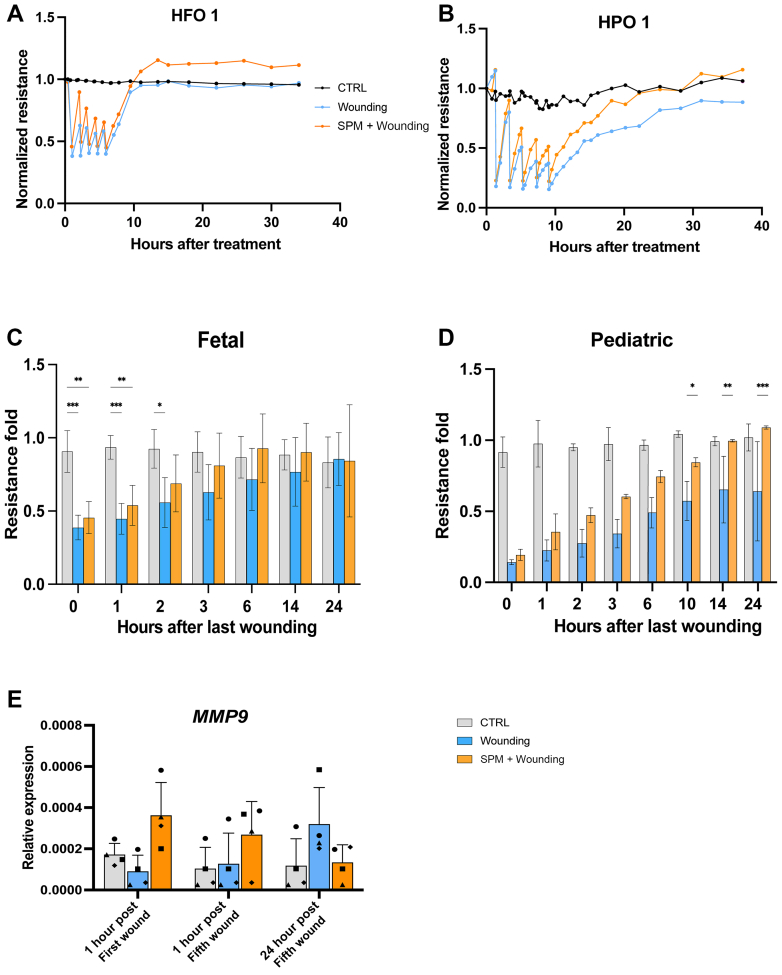
Effect of SPMs on TEER values of repetitively wounded 2D monolayers. Representative experiment of SPM restored the epithelial barrier of (A) an HFO and (B) an HPO monolayer. Baseline TEER values before wounding were 6262 ± 636 Ω·cm^2^ for HFO and 7301 ± 693 Ω·cm^2^ for HPO. TEER values were normalized to this baseline (time 0, immediately before SPM preincubation). Panels (C) and (D) show the mean normalized TEER values of 5 independent HFOs and 2 independent HPOs, respectively. Baseline absolute TEER values across all donors ranged from 4568–7223 Ω·cm^2^ for HFOs and 4513–8034 Ω·cm^2^ for HPOs. (E) Relative expression level of *MMP9* from 4 donors measured at 1 hour after the first wounding and 24 hours after the fifth wounding. Three technical replicates per donor were averaged prior to analysis, and values are mean ± SD of donor-level values in panels C, D, and E. ∗*P* < .05, ∗∗*P <* .01, ∗∗∗*P<* .001. A mixed-effects model with Tukey multiple comparisons test was used for C and D; Two-way repeated-measures ANOVA with Tukey post hoc test was used for panel E. CTRL, control group.

Organoids derived from 5 fetal and 5 pediatric donors were used to generate biological replicates. Each experiment was performed on at least 3 donors from each age group. Statistical analyses were performed using GraphPad Prism (version 9.5.0). For experiments with a single grouping variable and repeated-measures, and where data were not normally distributed (Figs. 1 and 2, Supplemental Figs. 3 and 4), the Friedman test was used, followed by Dunn post hoc test with Bonferroni correction. For experiments with unequal spacing or missing values (Fig. 3, C and D), a mixed-effects model was employed with the Tukey multiple comparisons test. For the experiments shown in Figure 3E, a 2-way repeated-measures ANOVA was performed to assess main effects and interactions, followed by a Tukey post hoc test for all pairwise comparisons. Results are presented as mean ± SD of biological replicates, and a *P* value <.05 was considered statistically significant.

## 3 Results

### 3.1 An SPM cocktail does not affect maturation of 3D HFO

Previously, we had interrogated the maturation status of organoid cultures isolated from fetal tissues up to 20 weeks of gestation and showed that across multiple passages HFOs retained an immature phenotype.^22^ Therefore, these cultures can be used to study gut conditions related to immaturity. Because external factors such as cytokines can influence the fetal to adult gut epithelial maturation,^22^ we first attempted to determine whether the SPM cocktail itself influences the maturation and differentiation of human fetal intestinal epithelium or not. We opted for treating HFOs with a cocktail of LxA4, RvD1, and RvE1 with concentrations of either 100, 200, or 500 nM each and monitored the expression levels of maturation and differentiation markers thereafter.

The differentiation and maturation status were investigated in cultures derived from proximal or distal intestinal segments independently because intestinal maturation can differ along the proximal-to-distal axis.^19^^,^^22^ HFOs generated from 3 independent donors treated with the SPM cocktail did not exhibit significant differences in the expression of the epithelial stem cell markers leucine-rich repeat-containing G-protein coupled receptor 5 or olfactomedin 4 in either the proximal or distal small intestine in comparison to the control group (Fig. 1, A and B). The expression of brush-border markers intestinal alkaline phosphatase, sucrase-isomaltose, and lactase, as well as the enterocyte marker fatty acid-binding protein 6, varied across different organoid cultures, with no significant differences observed following SPM treatment. Likewise, no significant changes were noted in the expression of goblet cell (mucin 2) and Paneth cell (lysozyme)*,* retinoic acid–inducible gene 1 *α*) markers, or defense marker polymeric immunoglobulin receptor (Fig. 1, C–J). Similar results were obtained in distal intestinal segments (Supplemental Fig. 3).

In summary, our findings suggest that the SPM cocktail has no significant impact on the stemness or differentiation of 3D HFOs and does not enhance the maturation of fetal intestinal epithelium in vitro.

### 3.2 An SPM cocktail does not resolve FL-induced inflammation in 3D HFO

To investigate the potential of the SPM cocktail to resolve inflammation, we first attempted to induce an inflammatory microenvironment in 3D HFO cultures. Independent donors were treated with LPS and FL, or a combination of both, for 24 hours. LPS and FL are bacterial products from *E coli* O111:B4 and *Salmonella* Typhimurium, respectively, known to induce inflammation by activating TLR receptors.^4^^,^^32^^,^^33^ Both treatments tended to induce the production of various inflammatory cytokines, for instance, IL1*α*, IL1*β*, TNF*α*, and IL8, by epithelial cells. FL alone was sufficient to elicit a similar response as a combination of LPS and FL, whereas LPS alone was less effective at inducing the production of IL1*α*, IL1*β*, IL8, and TNF*α* (Supplemental Fig. 4).

Because the cytokine and chemokine response to FL was the most robust, and no synergistic effect was observed with costimulation with LPS, we next assessed the potential of the SPM cocktail to resolve FL-induced production of inflammatory cytokines in 3D HFOs. FL led to mild upregulation of *TLR5* expression compared to control conditions, which could not be prevented by SPM pretreatment (Fig. 2A). In HFOs, FL-induced upregulation of *IL1α*, *IL1β*, and *IL8* by 4–7-fold, whereas the addition of the SPM cocktail did not modulate this effect (Fig. 2, B–D). FL induction of *IL10* was absent and not affected by SPM treatment (Fig. 2E).

Overall, our findings indicate that the SPM cocktail does not modulate FL- induced proinflammatory factors in 3D fetal organoids.

### 3.3 An SPM cocktail does exert some protective effect on 2D intestinal epithelial barrier stimulated with LPS and TNFα

Next, we explored the effect of an SPM cocktail on early life barrier function. 2D organoid monolayers were generated from 3D cultures using transwells.^22^ To induce a compromised barrier, the monolayers were initially treated with FL. However, even at high concentrations (500 ng/mL), FL treatment did not affect the TEER of fetal organoid monolayers (data not shown).

**Fig. 4:**
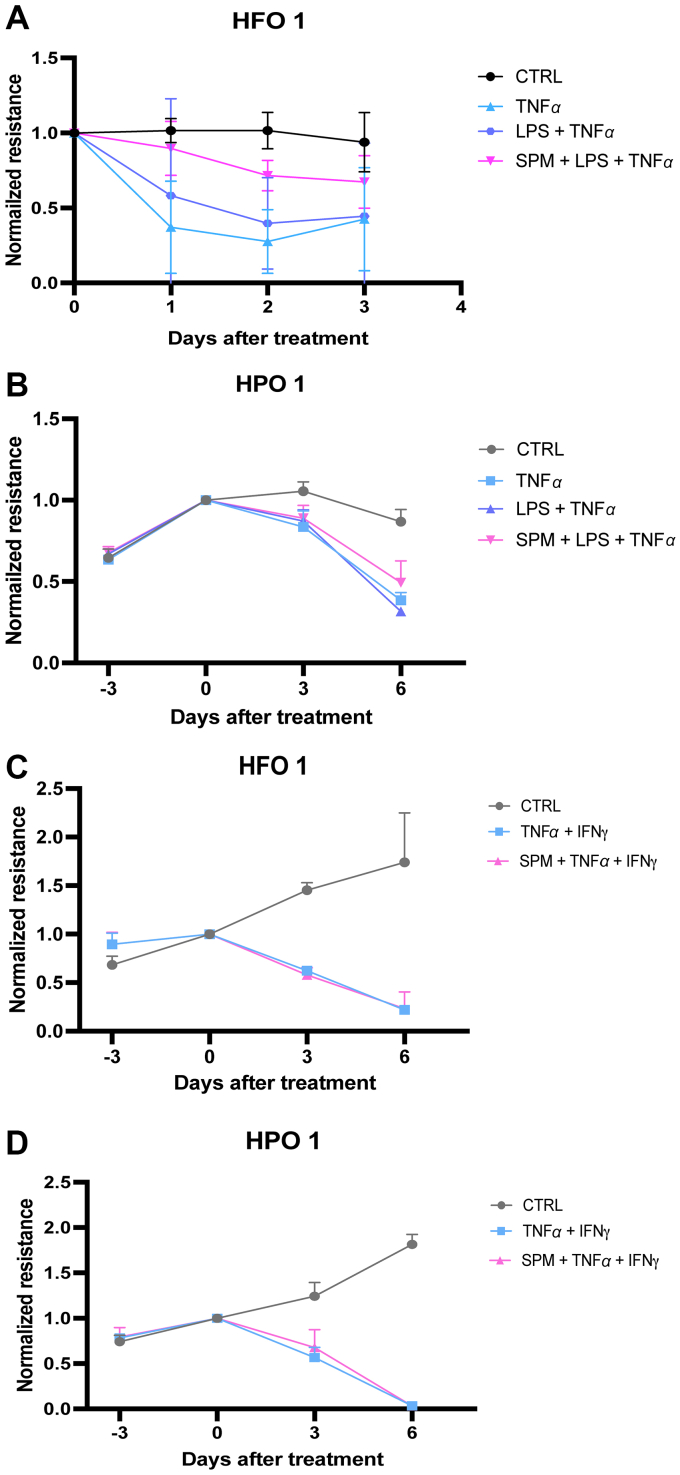
SPMs mitigated epithelial barrier dysfunction caused by inflammation induced by LPS and TNFα. TEER values of (A) HFO, and (B) HPO monolayers pretreated with SPM (200 nM) 1 hour prior to LPS (250 *μ*g/mL) and TNF*α* (400 ng/mL) stimulation for 3 days. Representative experiment of TEER values of (C) HFO, and (D) HPO monolayers pretreated with SPM (200 nM) 1 hour prior to stimulation with TNF*α* (200 ng/mL) and IFN*γ* (50 ng/mL) for 6 days. TEER values are normalized to baseline (before SPM preincubation). Data are presented as mean ± SD of 3 technical replicates per donor. These experiments were performed in individual donors and are therefore shown as representative examples; no statistical analysis was applied. CTRL, control group.

We next stimulated independent HFO and human pediatric intestinal organoid (HPO) 2D monolayers with TNF*α* (400 ng/mL) or a combination of TNF*α* (400 ng/mL) and LPS (250 *μ*g/mL) to induce epithelial barrier damage (Fig. 4, A and B). We noted variable response to TNF*α* treatment that was donor-dependent (data from other donors not shown). However, despite the donor-dependent effects of TNF*α* on HFO monolayers, we found that in the fetal donor who was responsive to TNF*α*, the SPM cocktail alleviated epithelial barrier dysfunction caused by LPS or TNF*α*/LPS-induced inflammation (Fig. 4A). In a different HPO donor, the effect of the SPM cocktail was not evident (Fig. 4B). Finally, we treated organoid monolayers from the same donors as shown in Figure 4, A and B with a combination of TNF*α* (200 ng/mL) and INF*γ* (50 ng/mL) (Fig. 4, C and D). The combination of these cytokines induced barrier damage, which was not alleviated by SPM treatment.

We also examined whether bacterial culture supernatants could induce barrier damage amenable to SPM rescue. However, *E coli* supernatant (25% v/v), either alone or in combination with mechanical wounding, did not significantly affect TEER values in either HFO or HPO monolayers, and no protective effect of SPMs was observed in this experimental paradigm (Supplemental Fig. 5). This result further underscores the specificity of SPM effects in the context of mechanical injury rather than broad anti-inflammatory or barrier-protective actions in our epithelial-only system.

### 3.4 An SPM cocktail restores the barrier function of repetitively wounded 2D monolayers

Given the donor-dependent effects of TNF*α* on organoid-derived monolayers, we used ECIS machine to monitor barrier function after inflicting cellular damage.^31^ The damage was inflicted by subjecting the cells to an electrical current, which created a wound in the epithelial barrier that can be healed by neighboring cells not subjected to the current. In the ECIS wounding and recovery assay, it was observed that it took approximately 2–3 hours for the organoid monolayer to recover to its initial TEER value. Because no significant difference in TEER recovery rates between the SPM-treated and control groups following a single electrical injury was observed, and during NEC, the epithelial barrier is exposed to continuous damaging stimuli, we applied 5 repetitive electrical injuries in total.

The organoid monolayers derived from HFOs and HPOs were incubated with the SPM cocktail starting 1 hour prior to wounding, whereas the controls were not. In all 5 fetal donors and 3 out of 5 pediatric donors that were tested, we observed that the TEER value recovered slightly better after the first wounding when pretreated with the SPM cocktail, with this difference becoming more pronounced over the 4 subsequent rounds of wounding (Fig. 3, A and B, Supplemental Fig. 6, A–E). Additionally, following the fifth wounding repetition, the TEER value of the SPM preincubated monolayer recovered more rapidly than that of the wounding-only control group, an effect maintained consistently across multiple organoid lines for 24 hours until the end of experiments (Fig. 3, C and D). These results were consistently observed across multiple donors (Supplemental Fig. 6, A–E).

We used RT-qPCR to test whether the genes related to various epithelial functions, such as apoptosis, proliferation, migration, and extracellular matrix remodeling, as well as epithelial barrier function and epithelial–mesenchymal transition, were affected by the SPM cocktail treatment in our assays. We observed that the SPM cocktail caused an increase in matrix metalloproteinase-9 (*MMP9*) expression 1 hour after the first wounding and then a faster decrease at 24 hours after the fifth wounding, whereas the wound-only group showed a delayed but sustained increase in expression (Fig. 3E). These data suggest that administration of the SPM cocktail affected *MMP9* expression dynamics in 2D HFO after wounding and enhanced the migratory capacity of wounded monolayers.

Overall, our findings indicate that preincubation with an SPM cocktail confers protective effects on the barrier function of epithelial monolayers after wounding.

## 4 Discussion

This study investigated the effects of an SPM cocktail on supporting the development of the intestinal barrier in early life and restoring its function under inflammatory conditions. To mimic early life, our study used 3D organoids and 2D organoid monolayers derived from human fetal and pediatric intestinal epithelial cells to study the effects of 3 specific SPMs, namely LxA4, RvD1, and RvE1. Our findings demonstrate that these SPMs had no influence on epithelial maturation or inflammatory responses in 3D organoid cultures challenged with FL and LPS. These SPMs showed potential to restore barrier function after TNF*α*-damage, but this effect was not reproduced in all donors tested. Moreover, in 2D monolayers exposed to both TNF*α* and IFN*γ*, these SPMs did not restore the compromised barrier function, indicating a limited capacity of SPMs under severe inflammatory conditions. However, these SPMs exhibited protective effects in 2D monolayers during repetitive mechanical injury, accelerating recovery after wounding and thereby maintaining barrier integrity.

Several prior studies have suggested that SPMs decrease inflammation, promote resolution, and trigger repair in challenged intestinal mucosa by inducing epithelial responses that function to regain mucosal homeostasis.^13^^,^^14^^,^^16^^,^^17^ In experimental colitis models, SPMs displayed beneficial effects in ameliorating colonic inflammation.^14^ However, most research on SPMs has focused on experiments in which the gut epithelium and immune system are fully developed, leaving a significant knowledge gap regarding the impact of SPMs on the inflammatory tone of gut epithelium in early life, when both the intestine and the immune system are less mature.

Our results show a lack of effect of SPMs on epithelial maturation. Previous studies have shown that LxA4, RvD1, and RvE1 can improve differentiation and barrier maturation of Caco-2 cells.^34^ However, early life organoid monolayers relate to intestinal tissue better than Caco-2 cells, which are derived from colorectal carcinoma tissue. Maturation-related genes in the early life phase were not significantly affected in our study. This indicates that SPM treatment has no effect on improving the maturation status of intestinal mucosa and therefore would have limited therapeutic potential in the setting of premature birth in the absence of inflammation.

In our study, we induced epithelial inflammation by treating 3D organoids with FL. In contrast to others who demonstrated an effect of SPMs on cytokine production, we did not observe an effect on FL-induced inflammation. These other studies showed that LxA4, RvD1, and RvE1 can inhibit the production of IL6, IL8, TNF*α*, and other cytokines.^35^, ^36^, ^37^, ^38^ However, the inflammation proresolving properties of SPMs are primarily described in immune cells themselves, and to the best of our knowledge, this is the first study to investigate the effect of SPMs on the inflammatory tone of gut organoids.^35^^,^^37^^,^^38^

We employed various approaches to impact barrier integrity, using TEER values as a measure of this integrity. FL was not able to affect TEER values in any of the donors tested. On the other hand, the response to TNF*α* treatment alone varied among donors and was not exacerbated by additional LPS treatment. We observed a reduction in TEER in only 1 out of 5 donors stimulated with LPS/TNF*α*. Previously, LPS/TNF*α* was successfully employed to induce inflammatory conditions in CaCo-2 monolayers.^39^^,^^40^ However, our study used fetal and young-infant epithelial cells, which may be more resilient to TNF*α*-induced damage. Interestingly, in the single fetal donor affected by TNF*α*, SPM treatment was able to restore TEER values to almost control levels. However, further research is needed to investigate the donor differences underlying this effect.

It is important to note that the high concentrations of cytokines (combination of 200 ng/mL rhTNF*α* and 50 ng/mL rhIFN*γ*) used in our barrier disruption experiments may have overwhelmed any protective effects of SPMs. Additionally, developmental differences in inflammatory signaling between fetal/pediatric and adult epithelium may influence SPM responsiveness. The immature state of TLR and cytokine receptor signaling in early life epithelium, combined with the absence of immune cells in our culture system, may explain why SPMs did not rescue high-concentration cytokine-induced barrier damage in our model despite showing efficacy in mechanical injury.

Conversely, severe inflammatory conditions induced by TNF*α*/IFN*γ* affected TEER in all tested donors. This aligns with other studies reporting that the complete loss of barrier integrity in these settings is associated with downregulation of tight junctions (claudin-1, occludin, and zonula occludens protein-1) and adherens junctions (E-cadherin) by TNF*α*/IFN*γ*.^41^ However, SPM treatment failed to restore barrier integrity under these conditions, suggesting that an SPM cocktail alone is insufficient to repair compromised barrier conditions. It is likely that the effectiveness of SPMs reported by others requires the presence of additional cues, possibly coming from other cell types beyond the epithelium, such as immune cells.^41^

We developed a protocol in which we repeatedly injured fetal and pediatric epithelial monolayers mechanically by applying an electrical current. Our observations revealed that SPMs could promote epithelial repair through enhancing migratory capacity, as evidenced by their potential to restore the injury of 2D monolayers, thereby allowing faster recovery of TEER values and affecting *MMP9* expression dynamics. This finding aligns with previous studies demonstrating the effect of SPMs on intestinal wound healing and restoring barrier function. Epithelial cells are key target cells for SPMs in the wound healing processes. For instance, increased migration of skin epithelial cells, which accelerates wound healing, has been observed after aspirin-triggered-RvD1 exposure, while LxA4 treatment promotes re-epithelialization in denuded mouse corneas.^42^^,^^43^ Quiros et al^13^ also demonstrated that localized delivery of RvE1 to intestinal wounds in vivo enhances mucosal repair by regulating epithelial proliferation. Our results extend to these findings, confirming that human epithelial cells in early life are also responsive to select SPMs, highlighting their therapeutic potential in neonatal contexts. However, it should be noted that our mechanistic interpretation regarding enhanced migration is based on MMP9 transcript levels, and we did not assess MMP9 protein expression or enzymatic activity in this study. Although mRNA changes often correlate with functional protein changes, future studies employing Western blotting or immunofluorescence microscopy would provide more direct evidence of MMP9 protein regulation by SPMs in this context.

The responsiveness of our fetal and pediatric organoids to SPMs suggests the presence of functional SPM receptors in early life intestinal epithelium. Although we did not systematically characterize receptor expression in the current study, previous work has demonstrated that intestinal epithelial cells express key SPM receptors, including formyl peptide receptor 2, which binds LxA_4_ and RvD1, and chemerin receptor 23, the receptor for RvE1.^44^, ^45^, ^46^, ^47^ However, it is important to note that our epithelial-only culture system limits our ability to assess immune cell-mediated mechanisms, which may be critical for the full spectrum of SPM effects observed in vivo. The absence of anti-inflammatory effects in our cytokine-challenged organoids may reflect this limitation because SPMs are known to exert potent effects on macrophages, neutrophils, and other immune cells that are absent from our model.

In conclusion, our findings suggest that LxA4, RvD1, and RvE1 have protective effects on epithelial barrier recovery in mechanically wounded monolayers, although their ability to reverse bacterial product- or cytokine-induced damage was not shown in our study. These results provide valuable insights into the therapeutic potential of SPMs in neonatal intestinal inflammation and barrier disruption, although clearly more research is needed.

## Conflict of interest

The authors declare no conflicts of interest.
