# Supplementary material for: Effects of specialized proresolving mediators on gut epithelial barrier in early life
Source: J Pharmacol Exp Ther. 2026 Jan 21;393(3):103815. doi: 10.1016/j.jpet.2026.103815 (PMC13084658; doi:10.1016/j.jpet.2026.103815)
Supplement: Supplementary Figures 1-6 [file mmc1.docx]

**Article title:**

Effects of specialized pro-resolving mediators on gut epithelial barrier in early life

**Authors:**

Jing Chen^1234^, Sarah Ouahoud^1,2^, Renee R.C.E. Schreurs^5^, Sander Meisner^1^, Jacqueline L.M. Vermeulen^1^, Manon E. Wildenberg^1^, Wouter J. de Jonge^1,2^ Johannes B. van Goudoever^234^, Tim G.J. de Meij^2,3,6^, Vanesa Muncan ‡ ^1,2^, Chris H.P. van den Akker ‡ ^2,3,4^

‡ These authors contributed equally to this work

**Journal:** *The Journal of Pharmacology and Experimental Therapeutics*

Supplemental Figure 1: Scheme of Bacterial Protein and Cytokine Stimulation of 2D organoid monolayers.

Supplemental Figure 2: Scheme of repetitive wounding and recovery assay.


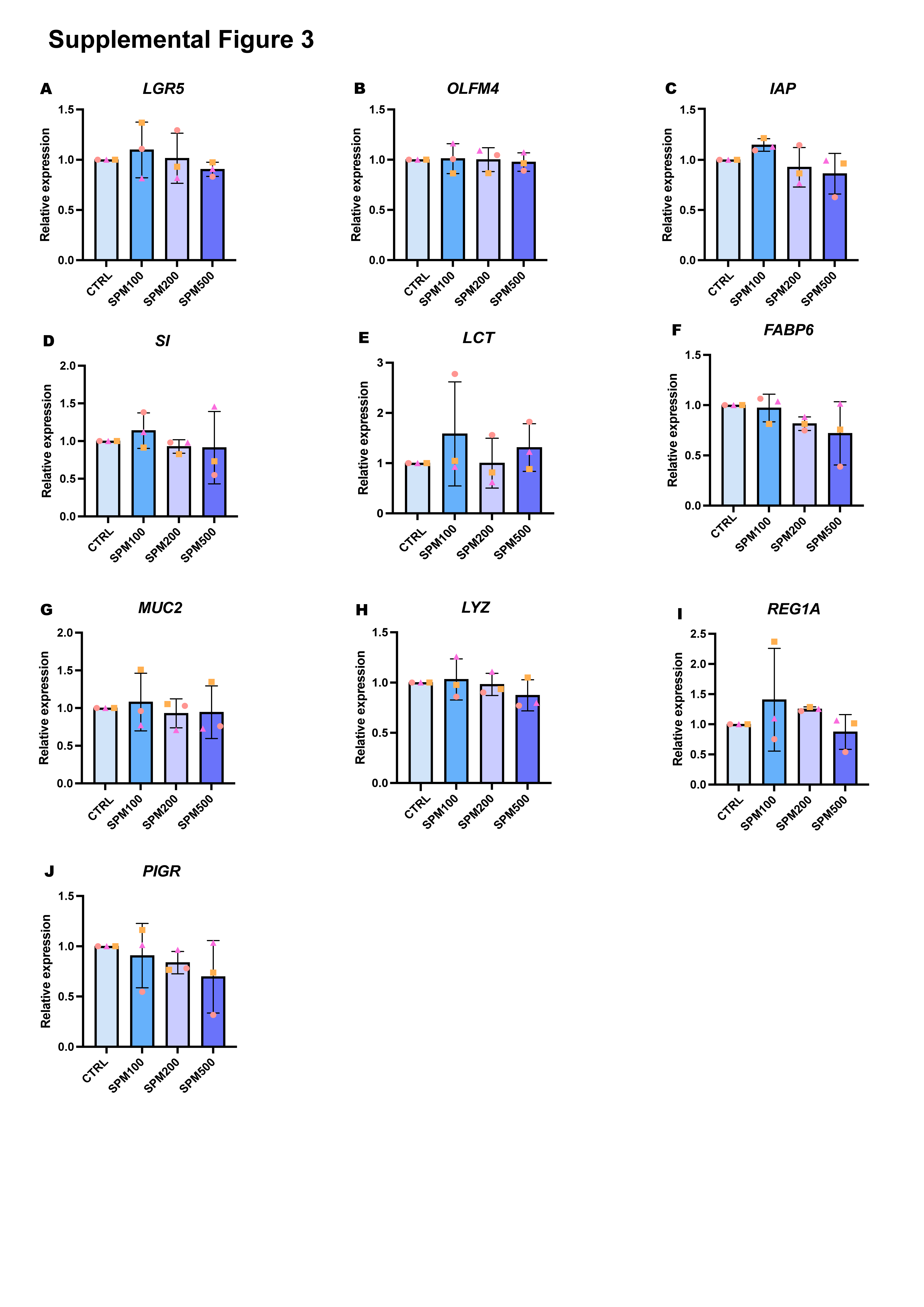


Supplemental Figure 3: HFO cultures retain immature features in terms of stemness, goblet and Paneth cells, absorption, and brush border enzyme markers expression when treated for 72 h with SPM.

RT-qPCR results of distal HFOs cultures of (A) *LGR5*, (B) *OLFM4*, (C) *IAP*, (D) *SI*, (E) *LCT*, (F) *FABP6*, (G) *MUC2*, (H) *LYZ*, (I) *REG1A*, (J) *PIGR*. Three technical replicates per donor were averaged prior to analysis. Values are mean ± standard deviation of n = 3 independent donors per condition. No significant differences were observed between treatment groups as determined by Friedman test, followed by Dunn's post hoc test with Bonferroni correction for comparisons to the control.


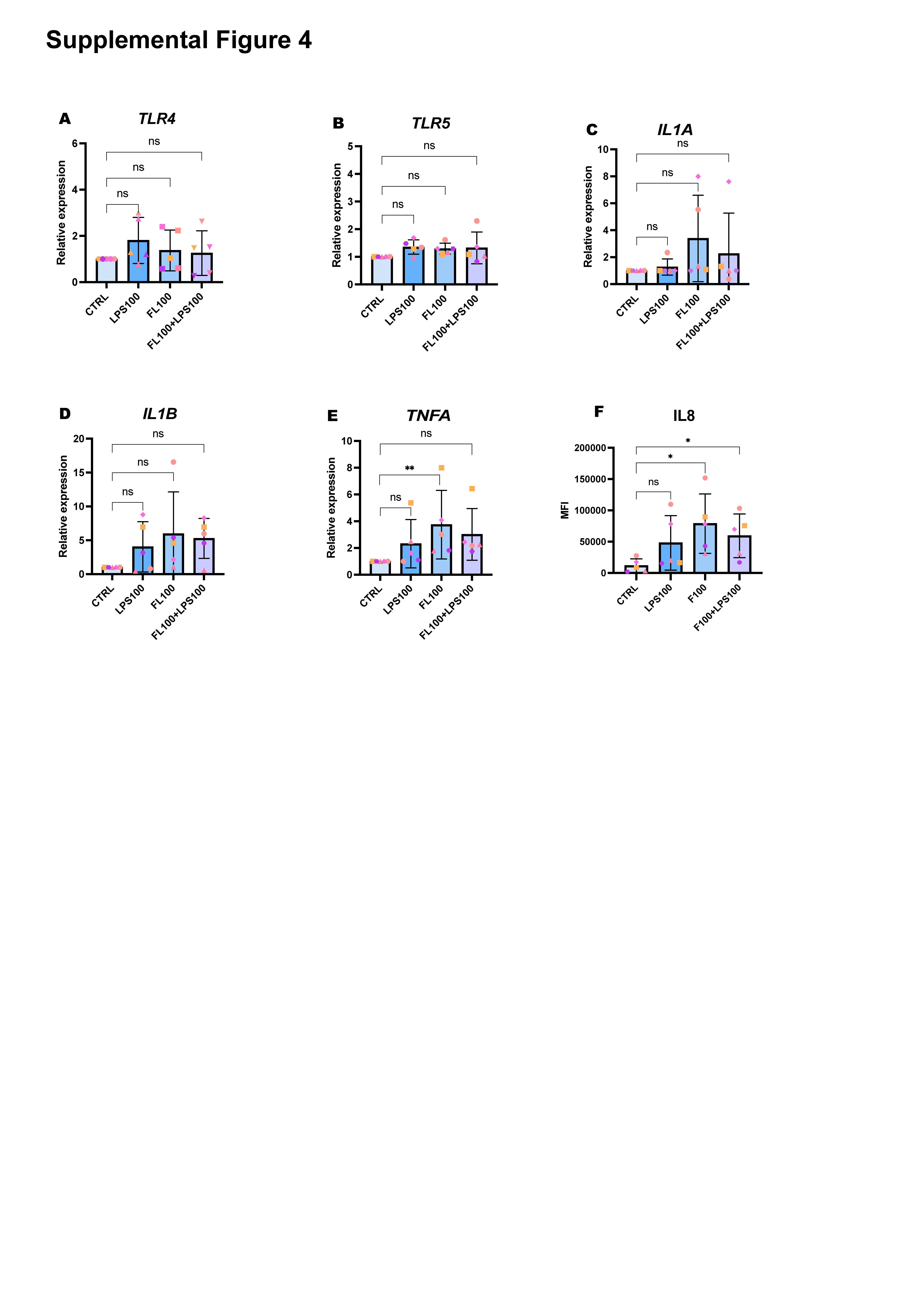


Supplemental Figure 4: Inflammatory response of HFO cultures to LPS or/and flagellin.

RT-qPCR results of HFO of gene expression level of (A) *TLR4,* (B) *TLR5*, (C) *IL1A*, (D) *IL1B*, (E) *TNFA,* and (F) CBA results of fluorescent intensity of IL8. Three technical replicates per donor were averaged prior to analysis. n =5 independent donors, distal intestine HFO of each donor displayed. Values are mean ± standard deviation. ∗*p* < 0.05, ∗∗*p* < 0.01, as determined by Friedman test, followed by Dunn's post hoc test with Bonferroni correction for comparisons to control.


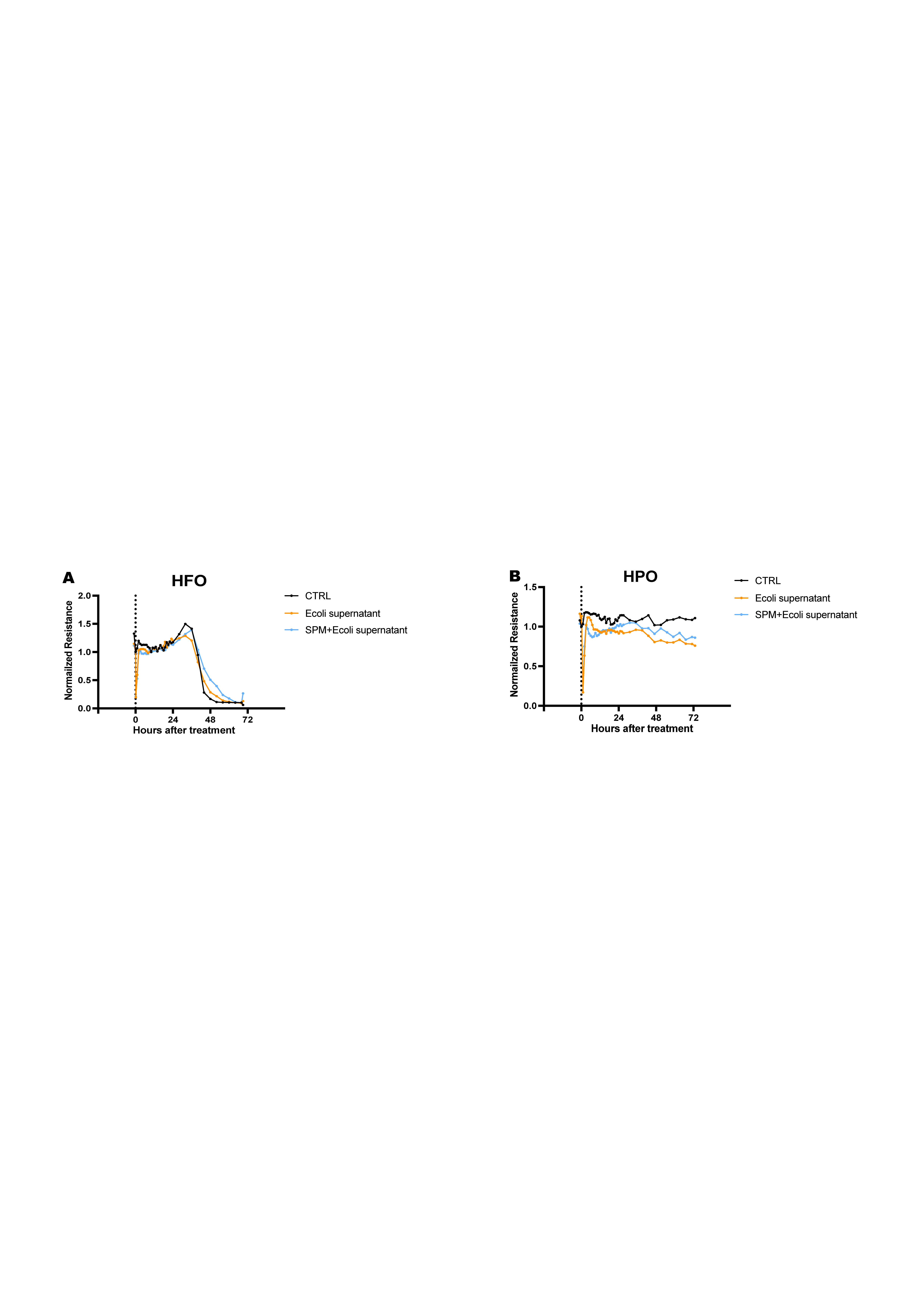


Supplemental Figure 5: Effect of E. coli supernatant on 2D monolayer TEER with or without SPM cocktail.

(A) HFO and (B) HPO monolayers were treated with 25% (v/v) *E. coli* culture supernatant with or without SPM cocktail (200 nM each), followed by a single electrical wounding 1 hour later (defined as time 0). TEER recovery was monitored for 72 hours. For each donor and condition, three technical replicates were averaged to generate a single TEER curve. Given the sample size (n=2 donors), no statistical testing was performed.


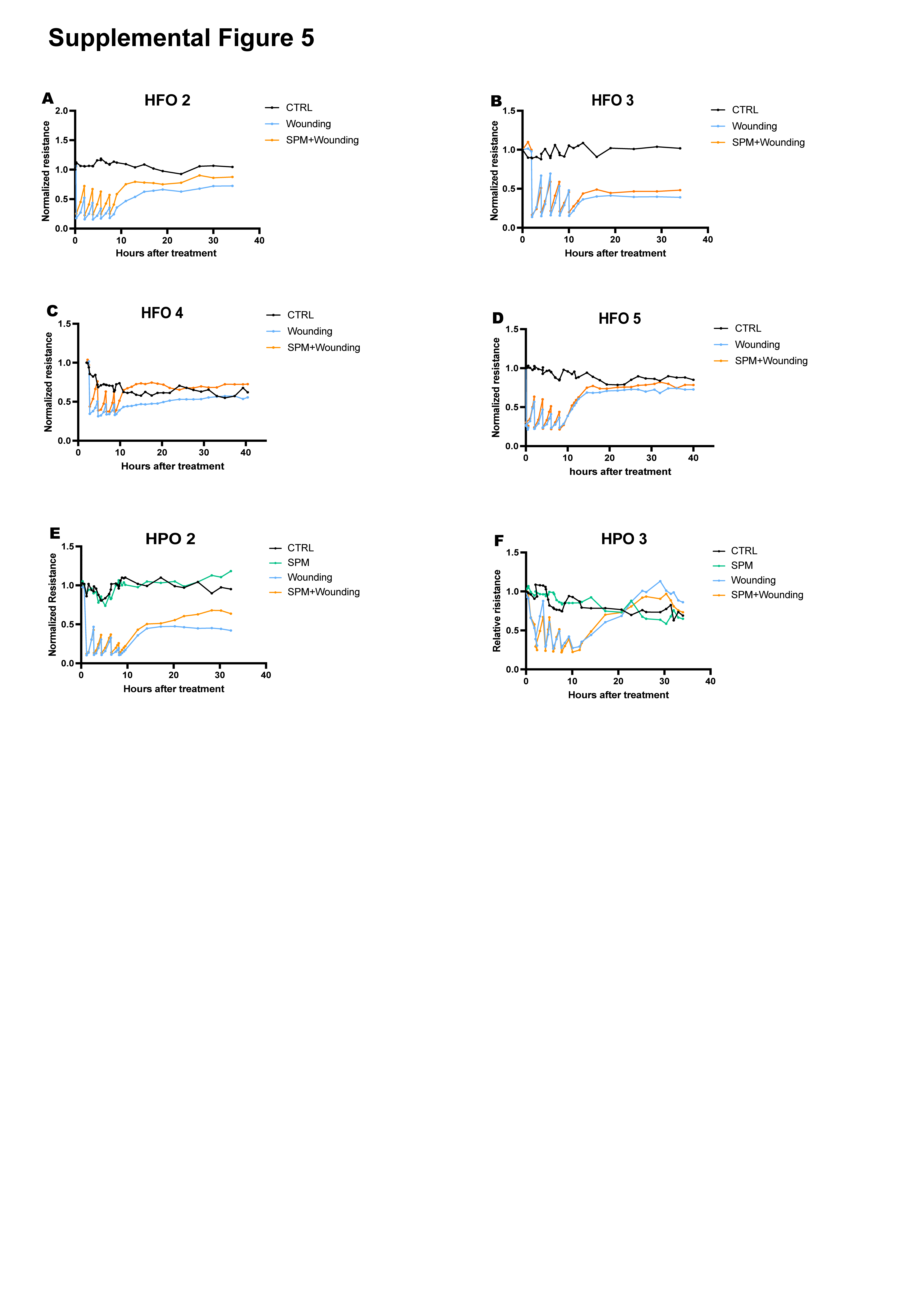


Supplemental Figure 6: Effect of SPMs on TEER values of repetitively wounded 2D monolayers.

SPMs restored epithelial barrier of (A-D) HFO and (E-F) HPO. TEER values are normalized to values before SPM pre-treatment. No statistical testing was performed.
